# Psychoactive pharmaceuticals as environmental contaminants may disrupt highly inter-connected nodes in an Autism-associated protein-protein interaction network

**DOI:** 10.1186/1471-2105-16-S7-S3

**Published:** 2015-04-23

**Authors:** Gaurav Kaushik, Michael A Thomas, Ken A Aho

**Affiliations:** 1 Department of Biological Sciences, Idaho State University, Stop 8007, 921 S 8th Ave, Pocatello, ID 83209-8007, USA

## Abstract

**Background:**

Most cases of idiopathic autism spectrum disorder (ASD) likely result from unknown environmental triggers in genetically susceptible individuals. These triggers may include maternal exposure of a fetus to minute concentrations of pharmaceuticals, such as carbamazepine (CBZ), venlafaxine (VNX) and fluoxetine (FLX). Unmetabolized pharmaceuticals reach drinking water through a variety of routes, including ineffectively treated sewage. Previous studies in our laboratory examined the extent to which gene sets were enriched in minnow brains treated with pharmaceuticals. Here, we tested the hypothesis that genes in fish brains and human cell cultures, significantly enriched by pharmaceuticals, would have distinct characteristics in an ASD-associated protein interaction network. We accomplished this by comparing these groups using 10 network indices.

**Results:**

A network of 7212 proteins and 33,461 interactions was generated. We found that network characteristics for enriched gene sets for particular pharmaceuticals were distinct from each other, and were different from non-enriched ASD gene sets. In particular, genes in fish brains, enriched by CBZ and VNX 1) had higher network importance than that in the overall network, and those enriched by FLX, and 2) were distinct from FLX and non-enriched ASD genes in multivariate network space. Similarly, genes in human cell cultures enriched by pharmaceutical mixtures (at environmental concentrations) and valproate (at clinical dosages) had similar network signatures, and had greater network importance than genes in the overall ASD network.

**Conclusions:**

The results indicate that important gene sets in the ASD network are particularly susceptible to perturbation by pharmaceuticals at environmental concentrations.

## Background

Autism is a complex neurobiological developmental disorder belonging to a group of conditions known as Autism Spectrum Disorder (ASD) [1,2]. ASD has an overall prevalence of approximately one case in every 50 children in USA [3], notably affecting four times as many males as females [4,5]. To date, several studies have reported ASD−associated genetic factors and have categorized them into 2 groups: rare variants (genes with low susceptibility and high penetrance) and common variants (genes with high susceptibility and low penetrance) [4]. These genetic factors, however, are responsible for only 2-3% of identified ASD cases [5,6].

In most other instances, studies suggest that ASD results from unknown environmental triggers acting on genetically susceptible individuals [6-9]. Susceptibility may be associated with gene variants [9] involved in biological pathways associated with ASD such as cell adhesion, synaptic vessel release, neurotransmission, and synaptic structure [6,10,11]. These biological pathways are inter-connected in a very complex manner [12]. However, it is unclear how environmental contaminants interact with or otherwise perturb ASD-associated biological pathways [12].

Today, genetically susceptible individuals may be exposed to combinations of 3000 synthetic chemicals via air, food and water [8]. Synthetic chemicals are generally categorized into two groups: pharmaceuticals and personal care products (PPCPs), and other industrial chemicals, such as organophosphate insecticides and organic solvents (e.g., ethyl alcohol) [8]. PPCPs include extensively used psychoactive pharmaceuticals [13], but also include bis-phenyl A in plastics, phthalates in cosmetics and household products, and known teratogenic pharmaceuticals [8]. In this study we focus on psychoactive pharmaceuticals that 1) may find their way to drinking water from clinical dosages excreted by patients, 2) are generally untreated by waste-water treatment plants [14], and 3) have sufficiently long half-lives [10] to eventually emerge in drinking water. Because many PPCPs are known to perturb neurological systems, exposure of a fetus to these contaminants by way of the pregnant mother's water consumption is a plausible environmental risk factor for neurological disorders like ASD [7,8].

In a previous study, we investigated psychoactive pharmaceuticals presented at very low concentrations in the environment [10,11]. Juvenile fathead minnows (*Pimephales promelas*) were exposed to fluoxetine (FLX), venlafaxine (VNX) and carbamazepine (CBZ) individually and in mixtures at environmentally relevant concentrations [11]. Using gene-class analysis [15], gene expression data indicated enrichment (significant up- or down-regulation) of gene sets associated with neuronal growth, regulation, and development in the juvenile minnow brains in response to psychoactive drug exposure [11]. Moreover, a significant behavioral change (number of turns, lateralization, distance travelled) was observed in fish exposed to pharmaceuticals. We sought to identify potential pharmaceutical-associated gene expression mechanisms underlying these responses. As a first step, we considered the interaction of pharmaceuticals within the underlying ASD-associated protein network. This approach has been used elsewhere to determine how altering expression of one or a few genes can influence biological pathways, ultimately contributing to complex phenotypes [16].

We found previously that fathead minnows exposed to PPCPs had a significantly altered behavioral phenotype compared to a non-exposed (control) group [11], and that PPCP-enriched gene sets represented biological pathways, which play a major role in neuronal systems. Thus, we predicted 1) that gene sets enriched by CBZ, VNX, FLX and MIX (all three) contained proteins that would be more interconnected than genes in the overall ASD-network, and 2) that gene sets enhanced by individual pharmaceuticals would have distinct network characteristics from each other.

To address these hypotheses, we constructed a protein-protein interaction (PPI) network of gene products known to be associated with ASD [17,18], and examined 1) genes enhanced by particular PPCPs in fish brains, and 2) genes enhanced by a PPCP mixture and valproate (VPA) in human neuronal cells. We note that VPA is known to induce ASD-like phenotypes in mice [19]. We sought to quantify patterns among gene sets enriched by PPCPs in both fish brains and human cell cultures by analyzing their network indices. Of particular interest was the identification of relationships between PCPP treatments and highly interconnected gene sets, as these are more likely to have profound effects on the functioning of the protein network because of their ripple effects on downstream proteins [20].

## Results

**Figure 1:**
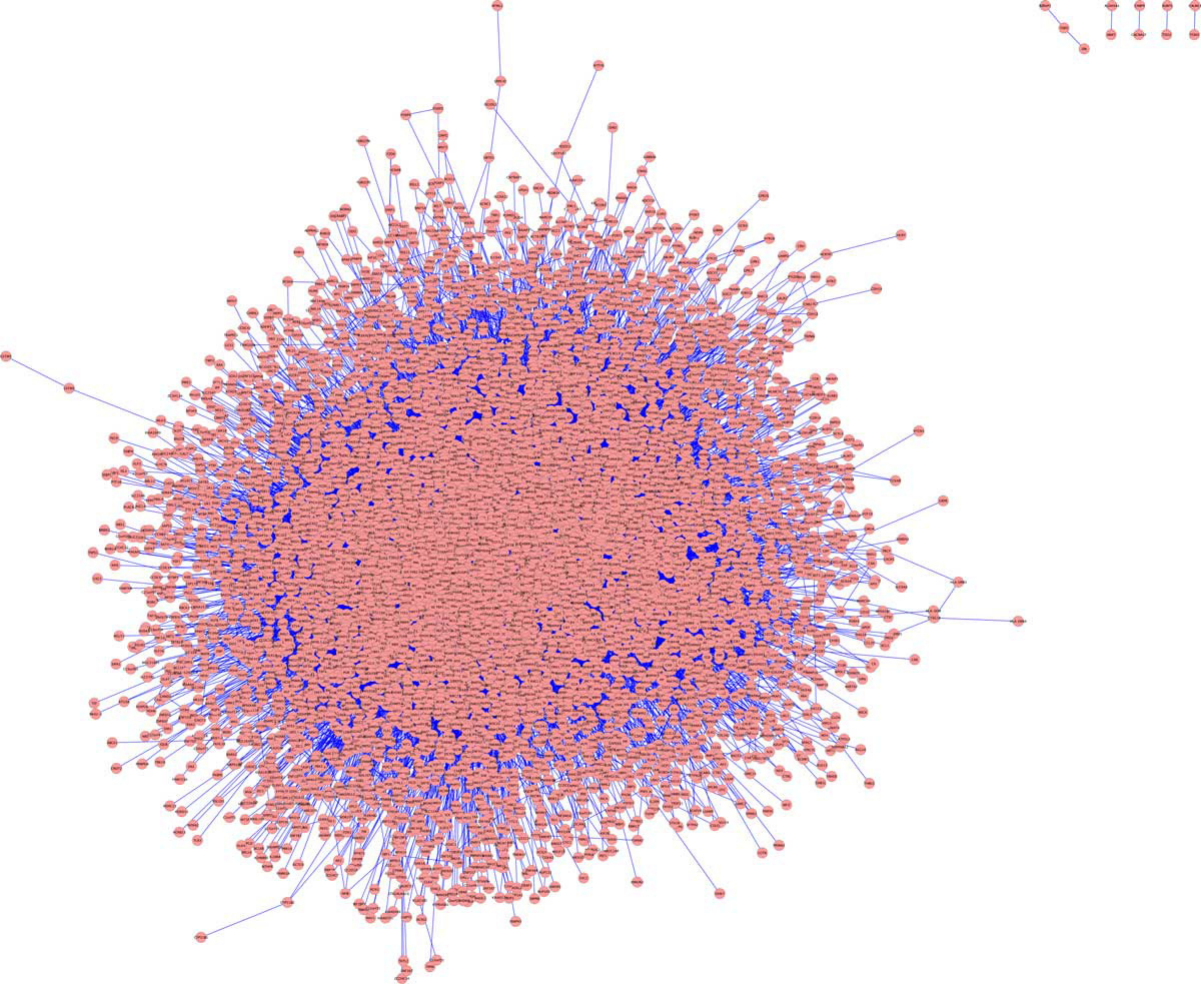
**Protein-protein interaction network associated with ASD**. The network was generated and visualized using Cytoscape v.2.8.3. Red dots represent nodes (or, proteins) and blue lines represent edges (or, connections among nodes).

We postulated that proteins within PPCP-enriched groups would have both distinct network characteristics from each other and higher levels of importance in the ASD protein-protein network. Support of this hypothesis would suggest that any dysregulation in the expression of PPCP-enriched proteins would result in large impacts to the network due to ripple effects on downstream proteins. To address this question, we generated an ASD protein-protein interaction network. The network consisted of 7212 nodes, with approximately 2000 primary neighbor proteins and 5000 secondary neighbor proteins (Figure 1). The average number of adjacent neighbors (average degree centrality; See additional file 1) for proteins in the network was 9.279, while the median degree centrality was 5.000. Additional summaries of network properties are listed in additional documents to this manuscript. (See additional file 1)

### In fish brains: Carbamazepine- and Venlafaxine- enhanced gene sets were more inter-connected in the ASD-network than both Fluoxetine- enhanced gene sets and the non-enriched portion of the network

**Figure 2:**
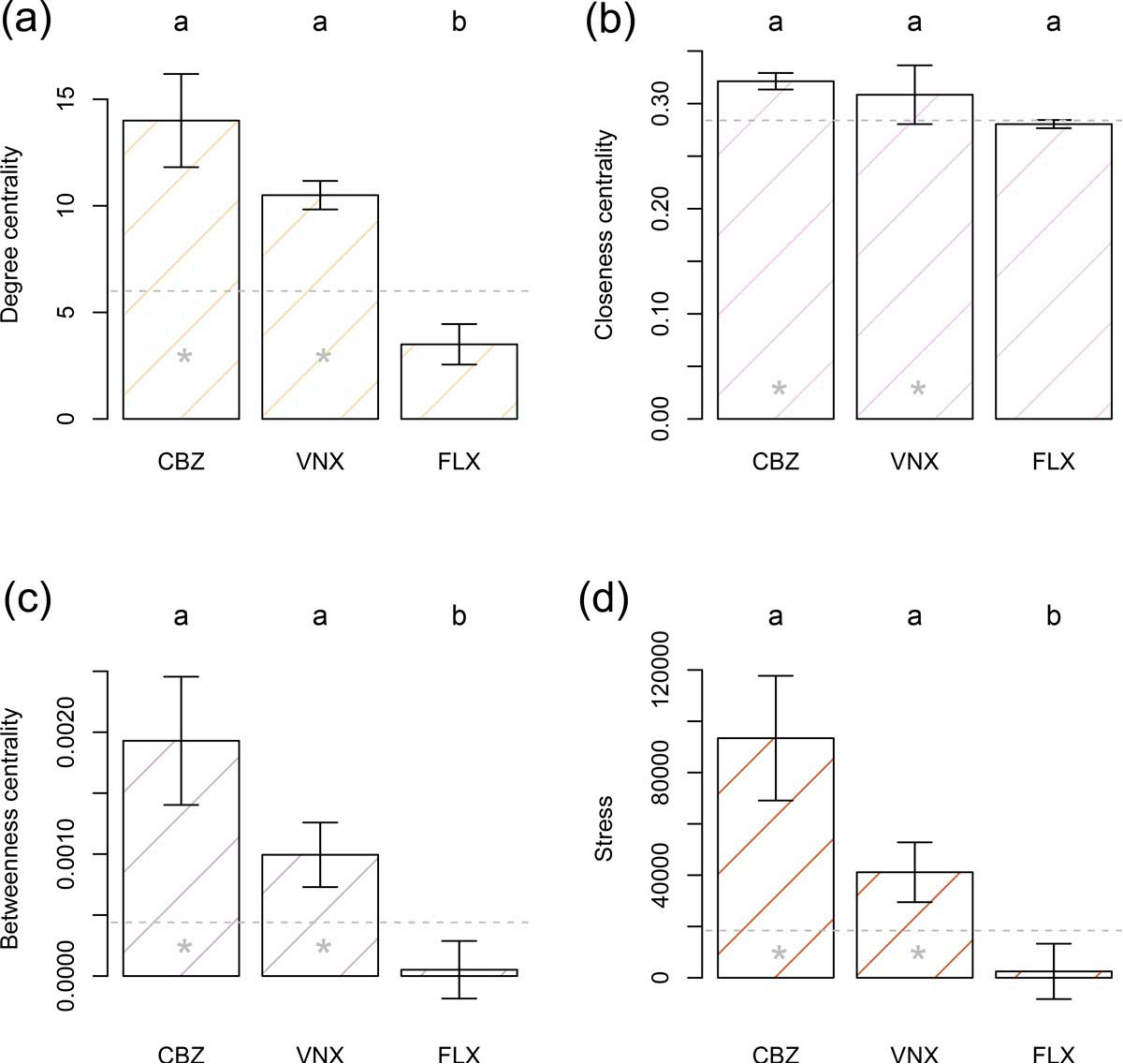
**Carbamazepine- and Venlafaxine- enriched gene sets were more inter-connected than Fluoxetine- enriched gene sets, and the overall network**. This figure compares network characteristics of gene sets enhanced by pharmaceuticals (CBZ, VNX, FLX) in fish brain tissue. Bars are pseudomedians (i.e., Hodges-Lehman estimators of location) whiskers are bootstrap standard errors of pseudomedians. Location measures with the same letter are not significantly different, using the BDM rank-based permutation procedure, after Holm's adjustment for simultaneous inference. Gray dashed horizontal lines are the pseudomedian values for the complete ASD network. Gray stars indicate that the treatment locations are significantly greater than the complete network pseudomedian, using Wilcoxon one-sample rank sum tests.

When considering fish brain cells, the VNX and CBZ groups had significantly higher location shifts (median and pseudomedian), compared to the overall network, for network indices widely deemed to be most important in identifying nodal importance (i.e., degree centrality, closeness centrality, betweenness centrality, and stress). The omnibus null hypothesis of equal location shifts for groups was rejected for all four of these measures allowing protected pairwise comparisons of groups [21]. In these comparisons the CBZ and VNX groups were statistically equivalent with respect to degree centrality, betweenness centrality and stress. The CBZ and VNX groups, however, had significant location shifts compared to the FLX group, and significantly higher locations than the overall ASD network (Figure 2). Strikingly, the FLX group degree centrality, closeness centrality, betweenness centrality, and stress locations were less than or equal to those of the overall ASD network (Figure 2).

**Figure 3:**
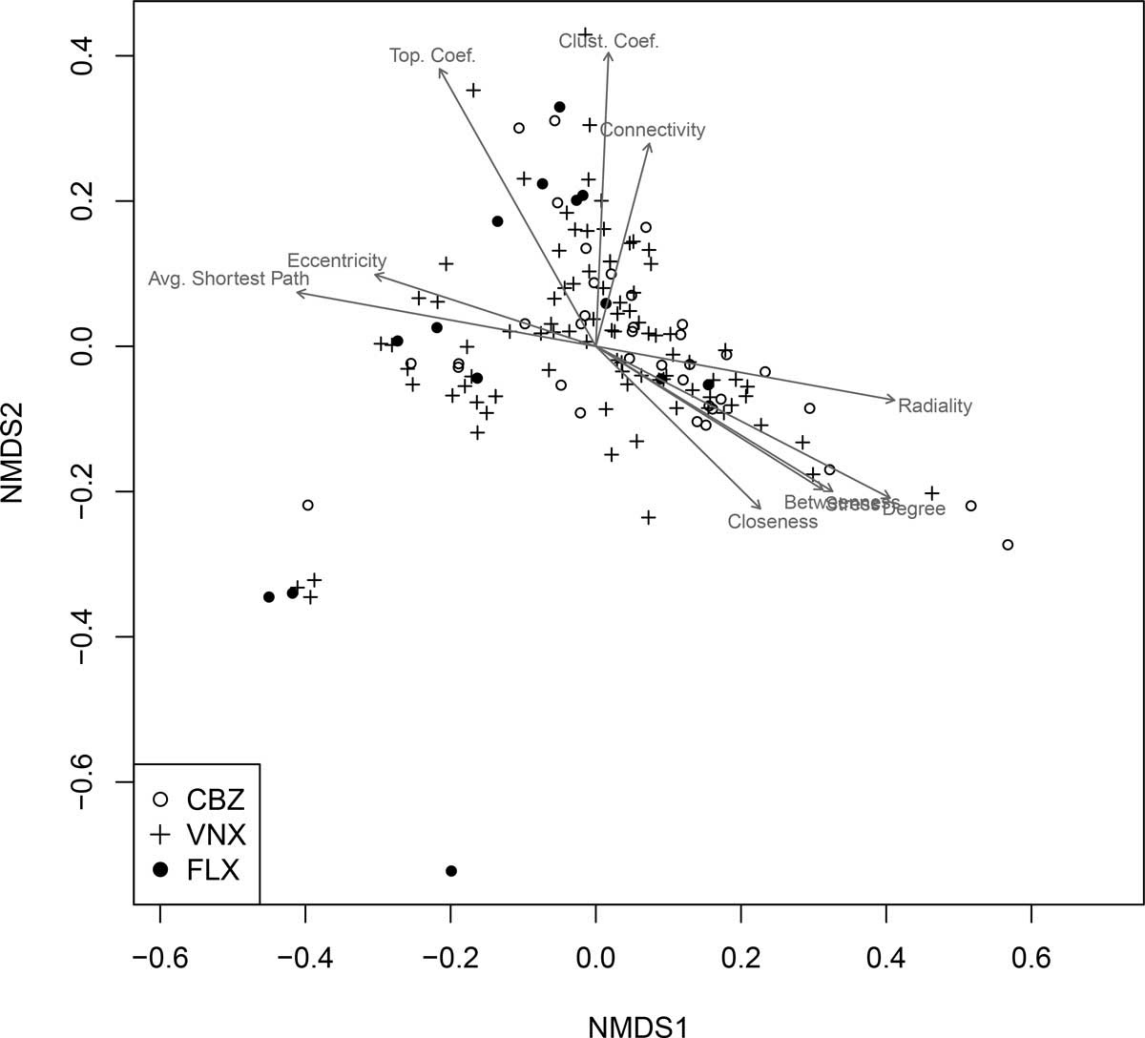
**Non-metric multidimensional scaling (NMDS) representation of significantly enhanced gene sets in fish brains**. This figure compares network characteristics of gene sets enhanced by pharmaceuticals in multivariate network space. Arrows within the scatterplot indicate the direction of most rapid increase for an indicated variable. Arrow length is scaled by the *R*^2 ^value of a multiple regression model in which the variable in question is the response and the NMDS axis scores are explanatory variables.

The collective network characteristics of pharmaceutical groups were considered using both permutation multivariate analysis of variance (PERMANOVA) and non-metric multidimensional scaling (NMDS). The fish brain cell genes of the non-enriched portion of the ASD network and individual pharmaceutical groups were significantly different in network space (*F*_3,1936 _= 3.68, *P *= 0.001). In pairwise PERMANOVA tests CBZ and VNX groups were indistinguishable from each other (*F*_1,123 _= 1.06, *P *= 0.352). Both of these groups, however, were significantly different from FLX (or essentially so) after adjustment for family-wise type I error (CBZ vs. FLX: *F*_1,48 _= 3.08, *P *= 0.052; VNX vs. FLX: *F*_1,101 _= 3.69, *P *= 0.023). Further, both the CBZ and VNX groups were statistically distinct from the non-enriched portion of the ASD network (CBZ vs. non-enriched: *F*_1,1832 _= 4.35, *P *= 0.020; VNX vs. non-enriched: *F*_1,1885 _= 4.70, *P *= 0.012), whereas the FLX group was not significantly different from the non-enriched fraction of the ASD-network (*F*_1,1811 _= 2.12, *P *= 0.136). The basis for these results is graphically evident in a non-metric multidimensional scaling (NMDS) dimension reduction of network space (Figure 3). We note that the CBZ group and VNX groups have similar network characteristics with high degree centrality, betweenness centrality, stress, and radiality. Conversely, the FLX group (more representative of non-enriched gene sets) had particularly small responses for these variables, but high values for eccentricity, average shortest path length and clustering coefficient.

### In human neuronal cells: mixture (CBZ, VNX, FLX) and Valproate - enhanced gene sets were more inter-connected than the overall network

To extend inference to human tissues, we extracted RNA from human neuronal cells treated with a PCPP mixture (CBZ, VNX, FLX) and valproate (VPA), and carried out transcriptome analysis. We then compared network characteristics of gene set groups, enriched by pharmaceuticals, both to each other, and to the overall network.

**Figure 4:**
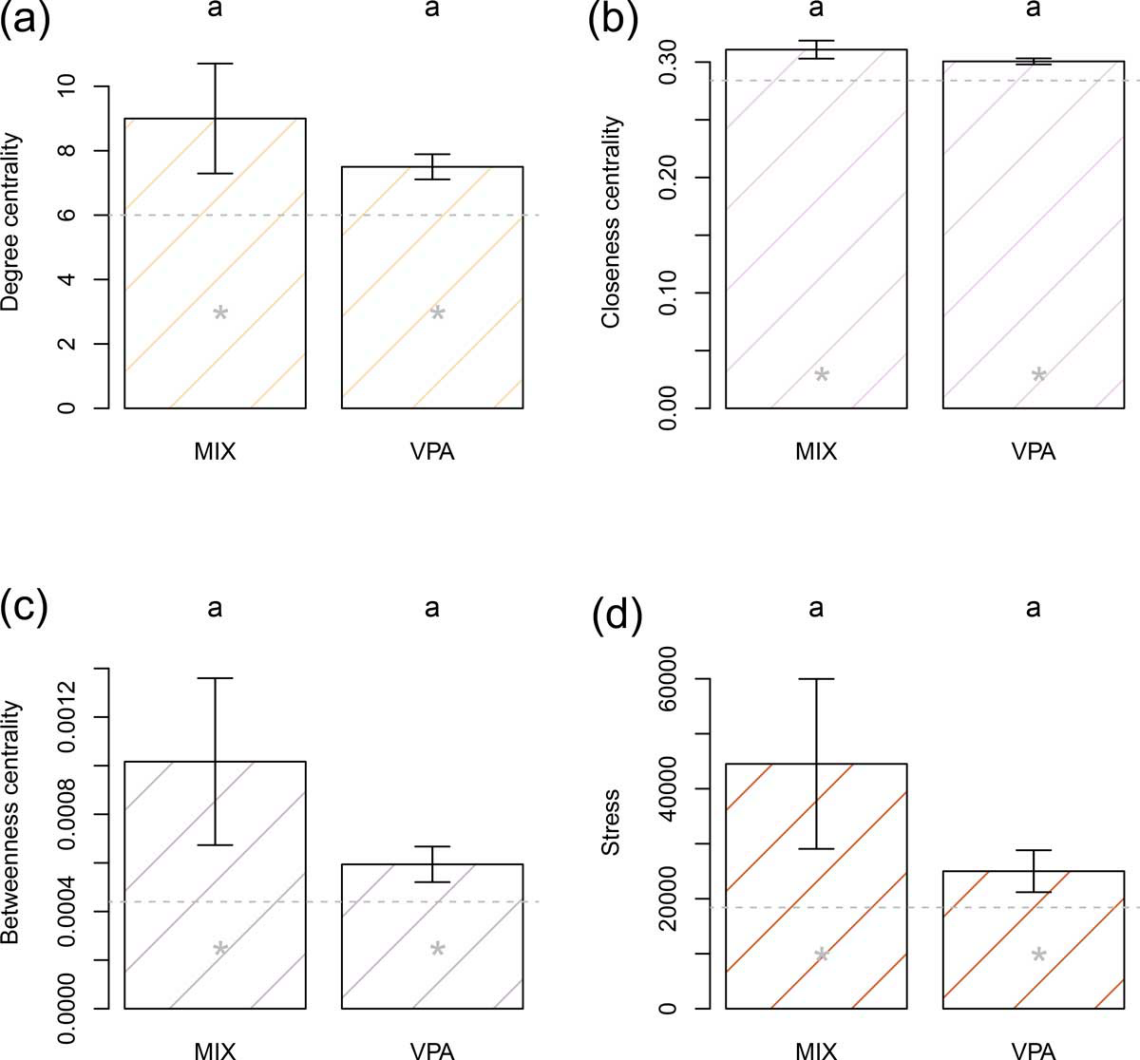
**PPCP MIX (CBZ, VNX, FLX) and Valproate (VPA) - enriched gene sets were more inter-connected than the overall network**. This figure compares network characteristics of gene sets enhanced by a mixture of pharmaceuticals (CBZ, VNX, FLX) and valproate (VPA) in human cell cultures. Bars are pseudomedians (i.e., Hodges-Lehman estimators of location) whiskers are bootstrap standard errors of pseudomedians. Location measures with the same letter are not significantly different, using the BDM rank-based permutation procedure, after Holm's adjustment for simultaneous inference. Gray dashed horizontal lines are the pseudomedian values for the complete ASD network. Gray stars indicate that the treatment locations are significantly greater than the complete network pseudomedian using Wilcoxon one-sample rank sum tests.

We found that both the mixture and VPA group locations were significantly higher than the overall network with respect to degree centrality, betweenness centrality, and stress. The mixture and VPA groups were, however, not statistically distinguishable from each other with respect to these measures (Figure 4).

**Figure 5:**
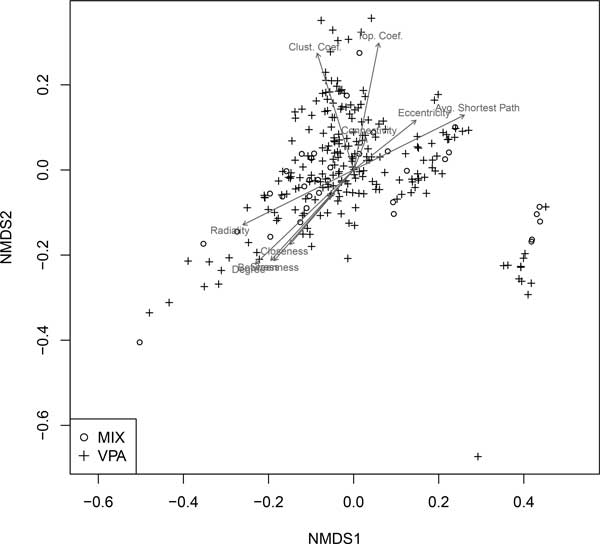
**Non-metric multidimensional scaling (NMDS) representation of significantly enhanced gene sets in human cell cultures**. This figure compares network characteristics of gene sets enhanced by pharmaceuticals in mixtures and valproate in multivariate network space. Arrows within the scatterplot indicate the direction of most rapid increase for an indicated variable. Arrow length is scaled by the *R*^2 ^value of a multiple regression model with the variable in question as the response variable and the NMDS axis scores as explanatory variables.

We rejected the omnibus null hypothesis that collective ASD network characteristics were equal for all groups, including the non-enriched portion of the ASD network (*F*_2,1690 _= 2.34, *P *= 0.017). Pairwise differences among groups, however, were not significant after adjustment for family-wise type I error (VPA vs. MIX: *F*_1,255 _= 2.10, *P *= 0.133; VPA vs. non-enriched: *F*_1,1876 _= 2.11, *P *= 0.132; MIX vs. non-enriched: *F*_1,1690 _= 2.85, *P *= 0.099). Characteristics of the VPA and mixture groups that may distinguish them from the overall ASD network are evident in Figure 5. We note both VPA and MIXTURE groups are split between genes that have higher degree centrality, betweenness centrality, closeness centrality, and radiality, and those--more representative of the non-enriched portion of the ASD network--that have smaller outcomes for these measures.

## Discussion

Researchers have identified many abnormal level of gene expression in the potential etiology of complex psychiatric disorders like ASD [4]. Due to many genetic factors like CNV deletions, mutations, allelic exclusion, or epigenetic silencing, the expression of synaptic proteins within the developing brain of a fetus might get altered [4]. This alteration in the level of synaptic proteins leads to the formation of abnormal neuronal circuits, which has been considered as one of the potential mechanism in ASD [4]. Other linked and association studies have found that these genetic factors are responsible for 2-3% of ASD cases [5,6], suggested the role to unknown environmental factors. Therefore, environmental factors are likely to be a major determinant because they may interact in combination with other genetic (pleiotropic and epistatic) factors causing varying ASD disease phenotypes [20,22].

We believed that psychoactive pharmaceuticals at environmental concentrations may dysregulate the expression of key synaptic proteins that further disturb the process of synaptogenesis. We carried out preliminary studies on juvenile fathead minnow fish exposed with Carbamazepine (100 μg/l), Venlafaxine (50 μg/l) and Fluoxetine (10 μg/l) at concentrations, which were detected in the surface waters in US [10,11]. Interestingly, along with significantly altered gene expression in exposed fish brains, we also observed a significant behavioral change in treated fish (number of turns, lateralization, distance travelled) [10,11]. Similar work has been done in zebrafish, which showed abnormal behavior after exposing them with valproic acid (anticonvulsant like carbamazepine) [16]. This suggested that psychoactive pharmaceuticals at environmental concentrations might be altering neuronal circuits by interacting with key genetic factors, thus caused different phenotypes in fish [4,11].

In the context of fish brain tissues our primary hypothesis was that human homologs of protein products of genes in synaptic cells, perturbed by exposure of pharmaceuticals, would have distinct ASD network parameters. To test this, we determined the extent to which protein products of genes from gene sets enhanced by individual pharmaceuticals in fish brains were interconnected in the ASD-associated protein-protein interaction network. As predicted, PPCP-enhanced sets had higher degree, closeness, betweenness and stress than the overall network. Further, we found that CBZ and VNX groups had higher degree centrality, closeness centrality, stress and betweenness centrality than FLX group, and were distinct from the FLX group and the non-enriched portion of the ASD-network in multivariate network space (Figure 2 &3). Interestingly, these distinctions were reflected by behavioral data in our previous study [11]. In this work, fish exposed to environmental concentrations of CBZ and VNX displayed more agitated behavior (e.g., number of turns, lateralization, distance travelled) than fish exposed to environmental concentrations of FLX [11].

To extend these findings to human tissues, we cultured and differentiated human SK-N-SH neuronal cells and carried out transcriptome analysis after exposing them with the mixture (CBZ, VNX, FLX) at environmental concentrations and valproate at a clinical dosage [23]. We then identified proteins groups significantly enriched in the presence of these pharmaceuticals, and quantified their importance in the ASD protein-protein network. We chose valproate (an anticonvulsant) as a treatment because prenatal exposure is associated with childhood autism [7]. Mixture and valproate groups had greater importance (e.g., higher degree, closeness, stress and betweenness) than the overall network (Figure 4 &5). We note that half of the genes from mixture group were also in the valproate group (although overlapping genes were not included in analyses). This suggests that the PCPP mixture would induce gene expression in cell cultures in a similar pattern to valproate.

Carbamazepine is a mood stabilizer and anticonvulsant used in conjunction with valproate to treat bipolar disorders and epilepsy [19,24]. CBZ and VPA block sodium channels, thus inhibit the epileptic effects in the brain [24]. CBZ is present at very low concentrations in the surface waters of United States [10,11], and may diffuse into groundwater [10]. Because the mixture group (which included CBZ) and valproate perturbed similar genes with relatively high network importance, we posit that 1) protein products of genes from both PCPP mixture and VPA serve as important nodes within the ASD-associated network, 2) enrichment effects of clinical doses of VPA are similar to those for environmental concentrations of pharmaceutical mixtures, and 3) gene dysregulation caused by PCPPs will have relatively profound effects on ASD protein network because these genes would effect more downstream proteins when perturbed [20].

## Conclusions

Genes connected to a large number of neighbors in the ASD-associated protein-protein interaction network may play an important role in neuronal growth, development, and regulation. We found that protein products from gene sets with enriched expression in fish brains and human neuronal cells, due to an exposure of psychoactive pharmaceuticals, were comparatively more inter-connected to other neighboring proteins than protein products of non-enriched gene sets. Thus, these genes are more likely to experience altered expression upon exposure to PPCPs, causing further dysregulation of the whole interactome due to a ripple effect.

## Methods

### Construction of ASD-associated network

A list of 304 ASD-associated genes was retrieved from the Autism Database (AutDB) [4,25], hereafter defined as the ASD gene set. To construct the network, we used the bioinformatics plugins for the Cytoscape visualization system, freely available from http://www.cytoscape.org/ as an open source java application [17].

We used the MiMi Cytoscape plugin [26] to create the ASD-associated network [27]. MiMi searched for protein interactions for query genes in HPRD protein database [27]. We examined all primary and secondary neighbors (i.e. neighbors of neighbors) in HPRD database against the ASD gene set. We then identified all primary and secondary protein interactions for the ASD gene set. (See additional file 2).

From our previous microarray study on fish brain tissue, we identified all gene sets that were significantly (*α *= 0.05) enriched by the psychoactive pharmaceuticals carbamazepine, venlafaxine and fluoxetine individually. We merged all single gene sets that were enriched by particular pharmaceuticals into separate groups, resulting in an enriched gene set for each of the pharmaceuticals. (See additional file 3).

In a parallel application, we cultured and differentiated human neuronal SK-N-SH cells with retinoic acid. After differentiation, we treated cells with a mixture treatment (CBZ, VNX, FLX) at environmental concentrations [28,29]. As a positive control, we also treated cells with Valproate at a clinical dose, 0.035 mM. We then carried out transcriptome analysis on treated cells and identified significantly (*α *= 0.05) enriched gene sets. Thus, we defined two groups: mixture (MIX) and valproate (VPA) containing all gene sets significantly enriched by the corresponding treatment [23] (See additional file 4).

### Network analysis with plug-ins and identifying parameters of gene sets

We used the popular Java-based freeware package Cytoscape to map the ASD protein network [17]. We used the Cytoscape plug-in, Network Analyzer [26], to summarize the nodes of the generated network for ten network measures (See additional file 1). To this network we applied a number of statistical approaches relatively novel in the analysis of protein networks including recently developed robust univariate and multivariate null hypothesis testing methods and high dimensional mapping procedures. (See additional file 5)

### Statistical approach

#### Univariate comparisons

Network data for most indices were characteristically strongly positively skewed, and often contained outliers (extreme values). This necessitated the use of robust procedures for hypothesis testing and estimation. For location (typical value) summaries we felt that the pseudomedian (Hodges-Lehman location estimator) was the most appropriate measure [30]. For a single set of observations, the pseudomedian is simply the median of all possible pairwise means. For a difference of two populations the pseudomedian is the median of all possible pairs of differences, and is an unbiased estimator of effect size from a Wilcoxon rank sum test [31].

Characteristics of treatments were considered in two ways. First, nodal network outcomes for particular indices were compared to the pseudomedian values of the complete network using one-sample Wilcoxon rank sum tests with upper tailed alternative hypotheses. Second, nodal network outcomes for particular treatment were compared to each other using Brunner-Dette-Munk (BDM) rank-based permutation tests [32]. BDM tests are robust to both non-normality and treatment heteroscedasticity [33]. The latter is not true for conventional rank-based permutation procedures (i.e., the Kruskal-Wallis test). In BDM comparisons an ominbus one way layout with three factor levels was used. If the null hypothesis of equal population shifts was rejected, then pairwise BDM tests comparing treatments were applied, and *P*-values for these tests were corrected for family-wise type I error using Holm's procedure [34].

To test the null hypothesis of identical network characteristics for the treatment groups, including the non-enriched portion of the ASD-network, we used permutation multivariate analyses of variance PERMANOVAs [35]. Steinhaus dissimilarity was used for the underlying resemblance matrix [36]. *P*-values were calculated using 10,000 permutations of the vector of treatment assignments with respect to the resemblance matrix. To depict patterns in multivariate network space we used nonmetric multidimensional scaling (NMDS; [37]). This method attempts to reduce discrepancies (stress) in a resemblance matrix and a mapping solution defined, in part, by a user-specified dimensional choice. We again used Steinhaus dissimilarity for the underlying resemblance matrix. Following the recommendations of Kruskal and Wish [38], our NMDS solutions were the lowest stress results from 20 randomized starting configurations.

All statistical analyses we conducted using the statistical package R [31]. In particular, nodal summary data were from Cytoscape using the package R-Cytoscape [39], and estimation procedures and hypothesis tests were run using R-base packages, and the packages asbio [40] and vegan [41].

## Competing interests

The authors declare that they have no competing interests.

## Authors' contributions

GK, KA and MAT designed and carried out the research. MAT provided the direction and guidance for the research. KA carried out analyses using R-programming and GK collected all the data and interpreted it after testing hypotheses. GK wrote the manuscript. All authors have read and approved the final manuscript.

## Supplementary Material

Additional file 1 **Description of network indices used in this paper**. This word file contained brief explanations of network indices. Click here for file

Additional file 2 ASD-associated PPI network Cytoscape file. Cytoscape compatible file of Core network is available at https://sites.google.com/a/isu.edu/aho/ This file is in *.cys *format, and will be opened using Cytoscape software. (Cytoscape software can be downloaded for free at http://www.cytoscape.org). Click here for file

Additional file 3 Complete gene data sets for fish and human cells (CBZ-enriched in fish brains, VNX-enriched in fish brains, FLX-enriched in fish brains, Mixture-enriched in human cells, Valproate-enriched in human cells). These files are also available at https://sites.google.com/a/isu.edu/aho/ Click here for file

Additional file 4 Complete data of gene sets for fish and human cells. Excel files containing a complete data of all gene sets (CBZ-enriched in fish brains, VNX-enriched in fish brains, FLX-enriched in fish brains, Mixture-enriched in human cells, Valproate-enriched in human cells). These files are available at https://sites.google.com/a/isu.edu/aho/ Click here for file

Additional file 5 Nodal summary statistics for gene set groups. The file contains summary statistics for all nodes in the network. This file is available at https://sites.google.com/a/isu.edu/aho/ Click here for file

## Declarations

The publication costs for this article were funded by an Institutional Development Award (IDeA) from the National Institute of General Medical Sciences of the National Institutes of Health under Grant #P20GM103408.

This article has been published as part of *BMC Bioinformatics *Volume 16 Supplement 7, 2015: Selected articles from The 11th Annual Biotechnology and Bioinformatics Symposium (BIOT-2014): Bioinformatics. The full contents of the supplement are available online at http://www.biomedcentral.com/bmcbioinformatics/supplements/16/S7.
