# Supplementary material for: Psychoactive pharmaceuticals as environmental contaminants may disrupt highly inter-connected nodes in an Autism-associated protein-protein interaction network
Source: BMC Bioinformatics. 2015 Apr 23;16(Suppl 7):S3. doi: 10.1186/1471-2105-16-S7-S3 (PMC4423768; doi:10.1186/1471-2105-16-S7-S3)
Supplement: Additional file 1 — Description of network indices used in this paper. This word file contained brief explanations of network indices. [file 1471-2105-16-S7-S3-S1.docx]

# Tables

## Table 1 - Description of network indices used in this paper

| 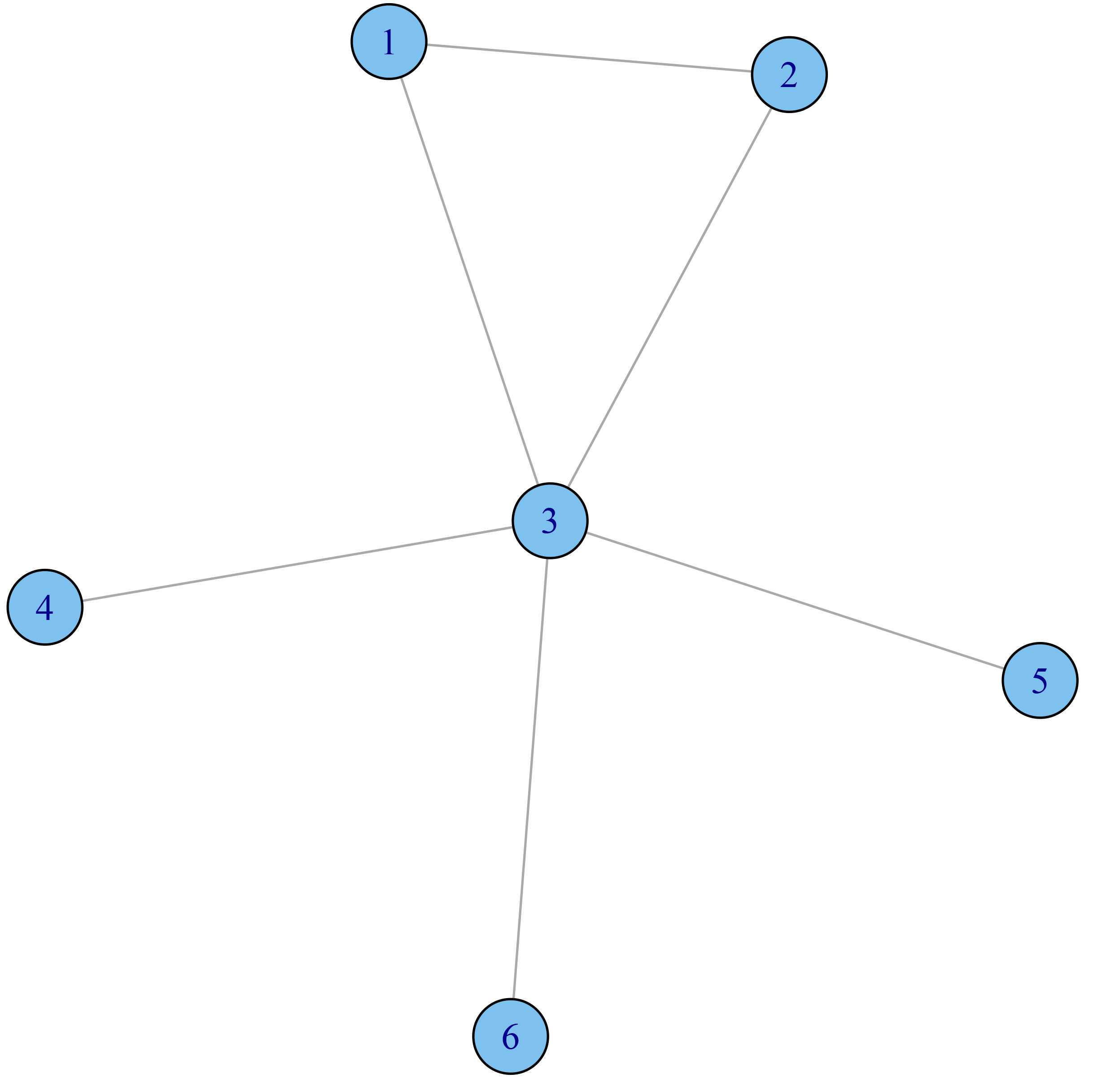  Example network with 6 nodes. |
| --- |
| **Degree centrality** of the *i*th node is the number of direct attachments of that node to other nodes. Thus node three has degree 5, and node six has degree 1.  **Closeness centrality** of a node is the inverse of the sum of the geodesic distances of the node to every other node in the network. $C_{C}(n_{i})=\left[\sum_{j=1}^{g} d(n_{i},n_{j})\right]^{-1}$   where *n_i_* indicates the *i*th node, *n_j_* indicates the *j*th node *j* ≠ *i*, *d* indicates geodesic distance, and *g* is the overall number of nodes.  Node three has closeness 1/(1+1+1+1+1)=1/5. Node six (and every other node) has closeness 1/(1 + 2 + 2 + 2 + 2) = 1/9.  **Betweenness centrality** of a node *i* is the number of “shortest geodesic paths” between node *j* and node *k* (*i* ≠ *j* ≠ *k*) that node *i* resides on.  **$C_{B}(n_{i})=\sum_{j<k} g_{jk}(n_{i})/g_{jk}$**  where *g_jk_* is the number of shortest geodesic paths connecting node *j* and node *k*, and *g_jk_*(*n_i_*) is the number of shortest geodesic paths that node *i* occupies. We note that *g_jk_* will always equal (*g*^2^ - *g*)/2 - (*g* - 1).  In the figure above, we have [(6^2^ − 6)/2] - 5 = 10 shortest pairwise paths, excluding those associated with the *i*th node. For node three these are (1,2) = 1, (5,6) = 2, (4,5) = 2, (1,4) = 2, (2,4) = 2, (1,5) = 2, (2,5) = 2, (4,5) = 2, (1,6) = 2, (2,6) = 2, (4,6) = 2, and (5,6) = 2. Node three occupies the shortest path in 9 of these. Thus, its betweenness centrality is 9/10. All other nodes have a betweenness centrality of zero.  The **clustering coefficient** (also called transitivity) of a node is the proportion of connections among its neighboring nodes that are realized compared to the number of all possible connections among the neighboring nodes. In the above Figure, node three has 5 neighbors. Thus, there are (5^2^ − 5)/2 = 10 possible connections between those neighbors. However only one set of neighbors actually communicate. Therefore the clustering coefficient of node three = 1/10 = 0.1. Nodes one and two each have 2 neighbors with (2^2^ −2)/2 = 1 possible connections. In both cases this connection is realized. Thus, the coefficient for nodes one and two is 1. The clustering coefficient of nodes four, five, and six is undefined because these nodes each have only one neighbor, preventing simultaneous consideration of communication between the nominal node and neighboring nodes, and communication among neighboring nodes.  The **topological coefficient** of a node measures the proportion of neighboring nodes shared with other nodes. Nodes with one or no neighbors are typically assigned a topological coefficient of 0. Node three has 5 neighbors, and 2 of these communicate. Therefore the topological coefficient for node three is 2/5 = 0.4.  The **average shortest path** for a node is simply the average of the shortest geodesic distances to every other node in the network. Node three has an average shortest path of (1 + 1 + 1 + 1 + 1)/5 = 1, nodes four, five, and six have an average shortest path of (1 + 2 + 2 + 2 + 2 + 2)/5 = 1.8, and nodes one and two have an average shortest path of (1 + 1 + 2 + 2+ 2)/5 = 1.6.  The **eccentricity** of a node is simply the largest geodesic distance of that node to all other nodes in the network. Node three has an eccentricity of 1, and all other nodes have an eccentricity of 2.  The **stress** of a node is the number of shortest paths passing through it. Thus, a node will have high stress if it is traversed by a large number of shortest paths. Again, we have 10 shortest paths. Node three is on 9 of these, so it has a stress of 9.  The **radiality** of a node describes its capacity to reach into a network and provide novel information. It has the form: $R(n_{i})=\frac{\sum_{j=1}^{g} [diam(G)+1-d(n_{i},n_{j})]}{(n-1)diam(G)}$   where *diam*(*G*) is the maximum geodesic distance in the graph. The distance of node three to all other nodes is 1, and the maximum geodesic distance in the graph is 2. Therefore the radiality of node three is [5(2 + 1 - 1)]/5 = 2. |

This table represents a brief explanation of the ten social network measures used in this paper. Examples given in the table are with respect to the embedded figure [42].
