# Supplementary material for: Psychoactive pharmaceuticals as environmental contaminants may disrupt highly inter-connected nodes in an Autism-associated protein-protein interaction network
Source: BMC Bioinformatics. 2015 Apr 23;16(Suppl 7):S3. doi: 10.1186/1471-2105-16-S7-S3 (PMC4423768; doi:10.1186/1471-2105-16-S7-S3)
Supplement: Additional file 5 — Nodal summary statistics for gene set groups. The file contains summary statistics for all nodes in the network. This file is available at https://sites.google.com/a/isu.edu/aho/ [file 1471-2105-16-S7-S3-S5.pdf]

Analysis of ASD protein-protein networks

October 14, 2014

Table 1: Network summary statistics for the ASD complete network

	Degree	Close.	Between.	Clust coef.	Top. coef	Avg. Short. path	Radiality	Eccentricity	Stress	Connectivity
n	7212.0	1948.000	1948.00000	1948.000	1948.000	1948.000	1948.0	1948	1948.0	1948.000
min	1.0	0.166	0.00000	0.000	0.000	1.000	0.4	1	0.0	1.000
max	248.0	1.000	0.06729	1.000	1.000	6.035	1.0	9	2941778.0	212.000
$\bar{x}$	9.3	0.303	0.00125	0.195	0.208	3.416	0.7	6	54694.1	37.122
median	5.0	0.299	0.00022	0.111	0.162	3.341	0.7	6	9953.0	29.250
HL-loc.	6.0	0.298	0.00044	0.146	0.185	3.379	0.7	6	18405.0	32.038
s	15.9	0.078	0.00407	0.246	0.170	0.545	0.1	1	176884.5	31.201
IQR	8.0	0.048	0.00093	0.267	0.197	0.548	0.1	1	36448.5	29.082
kurtosis	51.1	57.489	124.75551	3.553	1.514	4.379	4.4	18	116.0	8.870
skew	5.9	6.610	9.64041	1.953	1.228	0.189	-0.2	-2	9.3	2.481

Table 2: ASD network summary statistics for significantly enriched fish genes in the presence of CBZ

	Degree	Close.	Between.	Clust coef.	Top. coef	Avg. Short. path	Radiality	Eccentricity	Stress	Connectivity
n	116.0	48.000	48.00000	48.000	48.000	48.000	48.0	48	48.0	48.000
min	1.0	0.254	0.00000	0.000	0.000	2.352	0.7	5	0.0	8.200
max	244.0	0.425	0.06703	1.000	0.552	3.940	0.8	7	2618448.0	65.000
$\bar{x}$	20.4	0.322	0.00427	0.178	0.150	3.155	0.8	6	182075.3	32.112
median	11.0	0.325	0.00091	0.120	0.114	3.075	0.8	6	39500.0	32.604
HL-loc.	13.5	0.320	0.00175	0.136	0.120	3.146	0.8	6	80777.5	31.999
s	32.2	0.039	0.01114	0.208	0.129	0.378	0.0	1	446366.1	13.293
IQR	17.2	0.054	0.00313	0.134	0.092	0.542	0.1	0	151132.5	18.019
kurtosis	23.8	0.101	23.18651	8.967	1.831	-0.548	-0.5	-0	21.0	-0.327
skew	4.3	0.453	4.61645	2.813	1.556	0.120	-0.1	-0	4.4	0.325

Table 3: ASD network summary statistics for significantly enriched fish genes in the presence of VNX

	Degree	Close.	Between.	Clust coef.	Top. coef	Avg. Short. path	Radiality	Eccentricity	Stress	Connectivity
n	232.0	103.000	103.00000	103.000	103.000	103.000	103.0	103	103.0	103.000
min	1.0	0.196	0.00000	0.000	0.000	2.475	0.5	5	0.0	3.000
max	160.0	0.404	0.02903	1.000	0.769	5.090	0.8	8	1629878.0	207.000
$\bar{x}$	15.5	0.308	0.00184	0.158	0.179	3.303	0.7	6	83830.4	36.293
median	8.5	0.310	0.00061	0.108	0.145	3.221	0.8	6	28128.0	32.111
HL-loc.	11.0	0.309	0.00104	0.119	0.157	3.253	0.7	6	43609.0	32.565
s	20.8	0.038	0.00358	0.198	0.144	0.458	0.1	1	185122.9	27.124
IQR	16.0	0.046	0.00190	0.172	0.156	0.482	0.1	0	80376.0	24.440
kurtosis	15.1	0.467	33.37209	8.042	2.970	2.830	2.8	2	48.3	15.229
skew	3.3	-0.362	5.00705	2.570	1.581	1.352	-1.4	1	6.2	3.029

Table 4: ASD network summary statistics for significantly enriched fish genes in the presence of FLX

	Degree	Close.	Between.	Clust coef.	Top. coef	Avg. Short. path	Radiality	Eccentricity	Stress	Connectivity
n	41.0	16.000	16.00000	16.000	16.000	16.000	16.0	16	16.0	16.000
min	1.0	0.175	0.00000	0.000	0.000	1.000	0.5	1	0.0	1.000
max	33.0	1.000	0.00559	1.000	0.582	5.724	1.0	9	237990.0	108.000
$\bar{x}$	5.4	0.322	0.00068	0.221	0.214	3.549	0.7	6	31560.5	31.156
median	3.0	0.283	0.00003	0.107	0.186	3.535	0.7	6	1680.0	25.809
HL-loc.	3.5	0.284	0.00015	0.167	0.202	3.548	0.7	6	5176.0	26.004
s	7.5	0.186	0.00147	0.300	0.196	0.977	0.1	2	65393.6	28.864
IQR	4.0	0.054	0.00044	0.333	0.307	0.664	0.1	1	16947.0	24.383
kurtosis	8.5	13.852	8.76564	1.776	-1.076	3.559	3.6	7	6.6	2.656
skew	2.9	3.605	2.85047	1.523	0.452	-0.439	0.4	-2	2.5	1.575

Table 5: Network summary statistics for genes in human cell plates significantly enriched genes in the presence of a PCPP mixture

	Degree	Close.	Between.	Clust coef.	Top. coef	Avg. Short. path	Radiality	Eccentricity	Stress	Connectivity
n	158.0	70.000	70.00000	70.000	70.000	70.000	70.0	70	70.0	70.000
min	1.0	0.184	0.00000	0.000	0.000	2.403	0.5	5	0.0	5.000
max	244.0	0.416	0.03814	0.833	0.500	5.446	0.8	8	1793540.0	212.000
$\bar{x}$	15.5	0.305	0.00217	0.135	0.148	3.341	0.7	6	100337.4	40.938
median	7.0	0.308	0.00048	0.105	0.115	3.252	0.7	6	21803.0	31.552
HL-loc.	9.0	0.306	0.00087	0.111	0.134	3.291	0.7	6	40525.0	33.583
s	29.4	0.042	0.00639	0.155	0.117	0.511	0.1	1	291702.9	40.005
IQR	13.0	0.054	0.00182	0.176	0.136	0.573	0.1	0	82254.5	28.190
kurtosis	32.6	0.775	27.58532	6.624	0.542	3.687	3.7	2	26.7	9.864
skew	5.2	-0.094	5.19960	2.202	1.005	1.376	-1.4	1	5.1	2.961

Table 6: Network summary statistics for genes in human cell plates significantly enriched genes in the presence of VPA

	Degree	Close.	Between.	Clust coef.	Top. coef	Avg. Short. path	Radiality	Eccentricity	Stress	Connectivity
n	727.0	257.000	257.00000	257.000	257.000	257.000	257.0	257	257.0	257.000
min	1.0	0.184	0.00000	0.000	0.000	1.000	0.5	1	0.0	1.000
max	196.0	1.000	0.03814	1.000	0.654	5.446	1.0	8	1793540.0	212.000
$\bar{x}$	11.5	0.303	0.00152	0.176	0.185	3.374	0.7	6	65696.8	35.753
median	6.0	0.300	0.00030	0.111	0.153	3.334	0.7	6	14202.0	28.000
HL-loc.	7.5	0.301	0.00060	0.140	0.169	3.348	0.7	6	25426.0	31.000
s	19.4	0.058	0.00414	0.207	0.138	0.473	0.1	1	180533.8	29.630
IQR	9.0	0.046	0.00116	0.198	0.163	0.516	0.1	1	49698.0	26.307
kurtosis	35.3	83.750	40.35851	5.319	1.152	3.797	3.8	18	46.0	13.514
skew	5.2	6.934	5.87239	2.132	1.170	0.426	-0.4	-2	6.2	2.963
